# Aging Changes the Efficacy of Central Urocortin 2 to Induce Weight Loss in Rats

**DOI:** 10.3390/ijms24108992

**Published:** 2023-05-19

**Authors:** Dóra K. Kovács, Szimonetta Eitmann, Gergely Berta, Viktória Kormos, Balázs Gaszner, Erika Pétervári, Márta Balaskó

**Affiliations:** 1 Institute for Translational Medicine, Medical School, University of Pécs, Szigeti út 12., 7624 Pecs, Hungary; 2 Department of Medical Biology, Medical School, University of Pécs, Szigeti út 12., 7624 Pecs, Hungary; 3 Department of Pharmacology and Pharmacotherapy, Medical School, University of Pécs, Szigeti út 12., 7624 Pecs, Hungary; 4 Department of Anatomy, Medical School, University of Pécs, Szigeti út 12., 7624 Pecs, Hungary

**Keywords:** urocortin 2, obesity, aging, weight loss, metabolic rate

## Abstract

Middle-aged obesity and aging cachexia present healthcare challenges. Central responsiveness to body-weight-reducing mediators, e.g., to leptin, changes during aging in a way, which may promote middle-aged obesity and aging cachexia. Leptin is connected to urocortin 2 (Ucn2), an anorexigenic and hypermetabolic member of the corticotropin family. We aimed to study the role of Ucn2 in middle-aged obesity and aging cachexia. The food intake, body weight and hypermetabolic responses (oxygen consumption, core temperature) of male Wistar rats (3, 6, 12 and 18 months) were tested following intracerebroventricular injections of Ucn2. Following one central injection, Ucn2-induced anorexia lasted for 9 days in the 3-month, 14 days in the 6-month and 2 days in the 18-month group. Middle-aged 12-month rats failed to show anorexia or weight loss. Weight loss was transient (4 days) in the 3-month, 14 days in the 6-month and slight but long-lasting in the 18-month rats. Ucn2-induced hypermetabolism and hyperthermia increased with aging. The age-dependent changes in the mRNA expression of Ucn2 detected by RNAscope in the paraventricular nucleus correlated with the anorexigenic responsiveness. Our results show that age-dependent changes in Ucn2 may contribute to middle-aged obesity and aging cachexia. Ucn2 shows potential in the prevention of middle-aged obesity.

## 1. Introduction

Obesity is a major risk factor for numerous diseases. In 2016, at least 13% of the world’s population was obese and 39% was overweight [1]. Obesity continues to characterize human populations until old age; weight loss starts from age 75 [2]. In coming decades, the need for new medications preventing or treating obesity will rise.

Obesity is a multifactorial disease, which may also involve genetics, dietary excesses, sedentary lifestyle, unfavourable alterations of the microbiome, chronic stress, etc. [3,4]. Age-related regulatory alterations in energy balance may also contribute to middle-aged obesity, since other mammals and laboratory rodents also show weight gain during aging [5]. Previous research confirmed that age-related changes in the central anorexigenic and hypermetabolic responsiveness to leptin appear to promote middle-aged obesity [6]. The efficacy of other anorexigenic mediators, such as alpha-melanocyte-stimulating hormone (alpha-MSH), seems to show a similar age-related pattern, promoting middle-aged obesity [7]. To date, resistance (leptin) or side-effects (melanocortins) prevented the development of anti-obesity medication [8,9]. Thus, new targets need to be identified. Leptin increases the expression of other potential body-weight-reducing mediators in the hypothalamus, such as urocortin 2 (Ucn2) [10]. 

This peptide belongs to the corticotropin family. Of the four mediators of this peptide family, corticotropin-releasing hormone (CRH) was the first to be discovered, followed by urocortins 1, 2 and 3 [11,12,13,14,15]. These peptides can bind to type 1 and type 2 receptors of corticotropin-releasing hormone (CRH1R and CRH2R) [16]. The activation of CRH1R is responsible for numerous effects, including the activation of the hypothalamo–pituitary–adrenal axis involved in stress responses, and the increase in body temperature and heart rate. CRH1Rs also affect locomotor activity, anxiogenic and depressive behaviors, and even the health of the cartilage [17,18]. The anorexia elicited by CRH1Rs is attributed to anxiety [19]. On the other hand, CRH2Rs mediate anorexia, while inducing predominantly anxiolytic effects [17]. Moreover, they also induce hyperthermia [20,21] and locomotor activity [22,23]. Ucn2 binds selectively to the CRH2R [13]. Based on these characteristics, Ucn2 may be a worthy target of investigation in the search for anti-obesity drugs.

While the anorexigenic effects of Ucn2 are well-known [24,25,26], the changes in its efficacy during aging remain unknown. We aimed to investigate the role of Ucn2 in the changes in body weight during aging in a rat model. Therefore, we tested the age-related changes in the anorexigenic and hypermetabolic responsiveness to centrally applied Ucn2, along with changes in its capacity to reduce body weight. We also detected changes in Ucn2 mRNA expression in the paraventricular nucleus of the hypothalamus during aging. 

## 2. Results

### 2.1. The Effects of Urocortin 2 on the Food Intake and Body Weight

**Figure 1:**
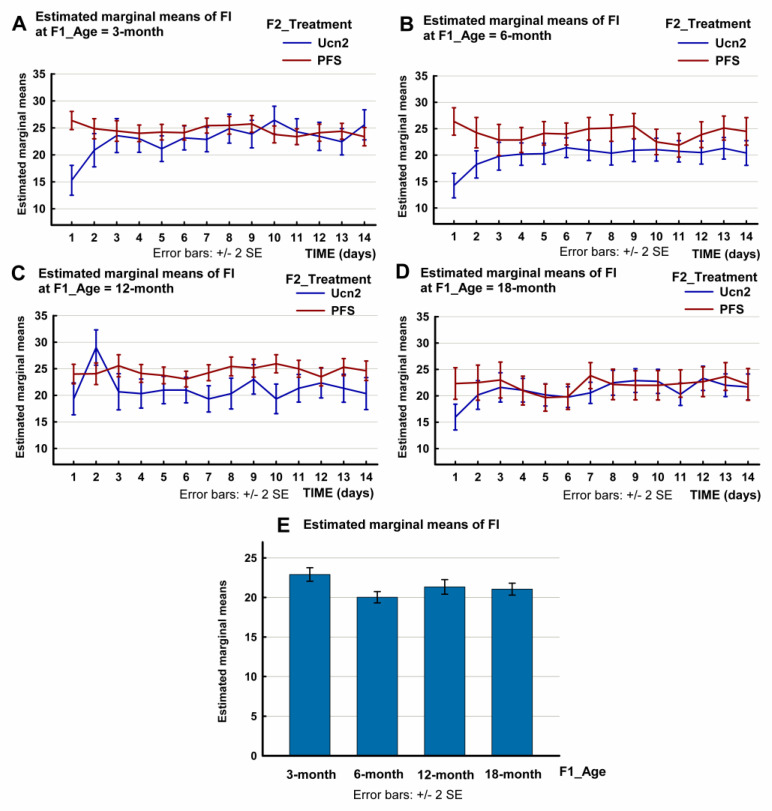
Daily food intake (FI) of male Wistar rats of different age groups (aged 3, 6, 12 or 18 months old) following an intracerebroventricular (ICV) injection of urocortin 2 (Ucn2) or pyrogen-free saline (PFS). The plots show the estimated marginal mean values of the FI of Ucn2-treated and PFS-treated control rats. (SPSS repeated-measures ANOVA, Profile Plots: food intake; time * F2_Treatment * F1_Age.) The bar chart (below) shows the estimated marginal mean 14-day food intake values of Ucn2-treated animals of various age groups. Data are also shown in Supplementary Tables S1 and S5. Number of animals per group: 3-month-old treated n = 7, 3-month-old control n = 8, 6-month-old treated n = 10, 6-month-old control n = 8, 12-month-old treated n = 6, 12-month-old control n = 8, 18-month-old treated n = 8, 18-month-old control n = 6).

With regard to food intake (FI) following the ICV injection of Ucn2 during the tested 14-day period, the multivariate test of the three-way repeated-measures ANOVA showed that, in addition to the time (F(13, 60) = 8.410, *p* < 0.0001) and the treatment (F(13, 60) = 12.456, *p* < 0.0001), the age factor (F(39, 178.421) = 2.372, *p* < 0.0001) affected the FI (Figure 1). An interaction was also shown by the test (time * treatment * age: F(39, 178.421) = 2.125, *p* < 0.0001). The effect size was large in all cases.

With regard to the within-subject effects, the Greenhouse–Geisser correction was applied. The effects of all the factors (time, treatment and age) were significant (Sums of Squares (SS) = 563,29, F(9.074, 653.332) = 8.004, *p* < 0.0001 for time, SS = 788.534, F(9.074, 653.332) = 11.205, *p* < 0.0001 for treatment and SS = 564.108, F(27.222, 653.332) = 2.672, *p* < 0.0001 for age). The effect size was medium in all three cases. There was a significant interaction between treatment and age (treatment * age: SS = 880.723, F(27.222, 653.332) = 4.172, *p* < 0.0001).

Between-subject effects were significant only for the treatment (SS = 1504.309, F(1, 72) = 15.218, *p* < 0.0001). The plots showing the estimated marginal mean values are shown in Figure 1 and in Supplementary Tables S1 and S5. 

We also compared the FI of the Ucn2-treated rats with that of control animals for all the groups and within each age group. This analysis also showed that the decrease in FI varied with aging during the tested 14-day period (F(7, 59) = 4.115, *p* = 0.001, Supplementary Figure S1 and Table S6). In the 3-month-old group, this decrease lasted for 9 days (repeated-measures ANOVA *p* = 0.02), in the 6-month-old group, the decrease remained significant throughout the 14-day observation period (*p* = 0.003), in the middle-aged, 12-month-old group, the Ucn2 failed to induce anorexia (*p* = 0.116), and in the 18-month-old group, the Ucn2-induced anorexia for 2 days (*p* = 0.024) (Supplementary Figure S1 and Table S6).

**Figure 2:**
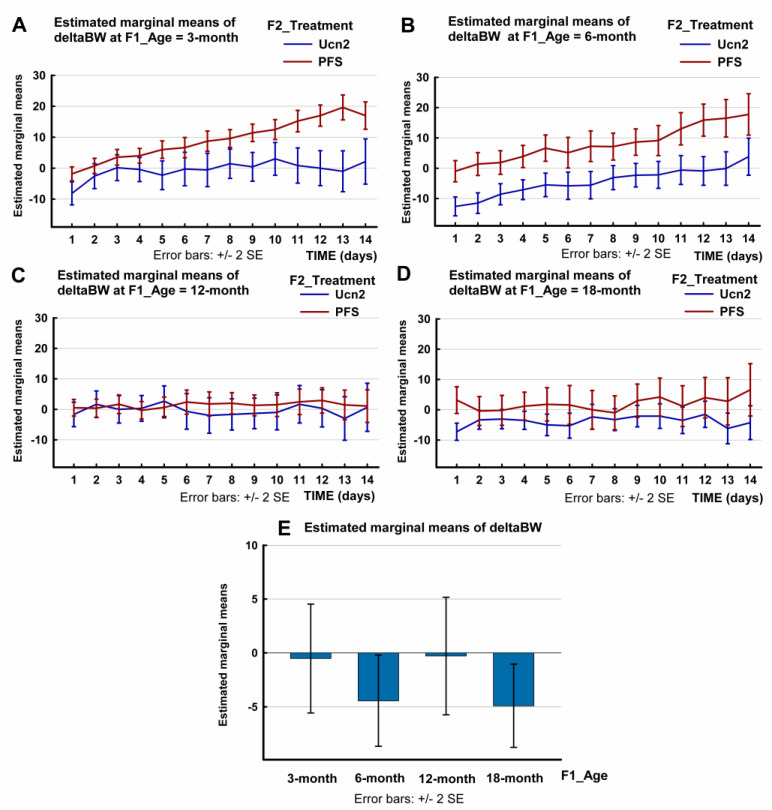
Changes in body weight (ΔBW) of male Wistar rats of different age-groups (aged 3, 6, 12 or 18 months) following an intracerebroventricular (ICV) injection of urocortin 2 (Ucn2) or pyrogen-free saline (PFS). The plots show the estimated marginal mean values of the ΔBW of Ucn2-treated and PFS-treated control rats. (SPSS repeated-measures ANOVA, Profile Plots: Body weight change; time * F2_Treatment * F1_Age). The bar chart (below) shows the estimated marginal mean 14-day ΔBW values of Ucn2-treated animals of various age-groups. Data are also shown in Supplementary Tables S2 and S5. Number of animals per group: 3-month-old treated n = 7, 3-month-old control n = 8, 6-month-old treated n = 10, 6-month-old control n = 8, 12-month-old treated n = 6, 12-month-old control n = 8, 18-month-old treated n = 8, 18-month-old control n = 6).

Concerning the change in body weight (ΔBW) following the ICV injection of Ucn2 during the tested 14-day period, the multivariate test of the three-way repeated-measures ANOVA showed that, in addition to the time (F(13, 59) = 7.045, *p* < 0.0001) and the treatment (F(13, 59) = 4.101, *p* < 0.0001), the age factor (F(39,175.459) = 2.119, *p* < 0.05) affected the weight loss (Figure 2 and in Supplementary Tables S2 and S5). There was no significant interaction between the treatment and age (time * treatment * age: F(39,175.459) = 1.419, *p* = 0.067). The effect size was large in all cases. 

With regard to the within-subject effects, the Greenhouse–Geisser correction was applied. The effects of all the factors (time, treatment and age) were significant (SS = 5798.107, F(3.490, 247.767) = 21.184, *p* < 0.0001 for time, SS = 1148.376, F(3.490, 247.767) = 4.196, *p* < 0.05 for treatment and SS = 4415.424, F(10.469, 247.767) = 5.377, *p* < 0.0001 for age). The effect size was strong for time and age and weak for treatment. There was no significant interaction between time, treatment and age (time * treatment * age: SS = 1188.837, F(10.469, 247.767) = 1.448, *p* = 0.156). 

Between-subject effects were significant for both treatment (SS = 14029.675, F(1, 71) = 40.510, *p* < 0.0001) and age (SS = 4273.653, F(3, 71) = 4.113, *p* < 0.051). There was also a significant interaction between treatment and age (treatment * age SS = 3841.219, F(3,71) = 3.697, *p* < 0.05). The plots showing the estimated marginal mean values are shown in Figure 2 and in Supplementary Tables S2 and S5.

We also compared the ΔBW of the Ucn2-treated rats with that of control animals for all the groups and within each age group. This analysis also showed that the decrease in BW varied with aging during the tested 14-day period. The change in the BW of young adult (3-month) rats showed significant difference from that of controls for 5 days (*p* = 0.01). The Ucn2-induced decrease in BW in the 6-month group exceeded that of controls throughout the observed 14-day period (*p* < 0.0001). No BW change was detected in the middle-aged 12-month group (*p* = 0.82), while repeated-measures ANOVA indicated that the curve of Ucn2-induced BW change in the oldest 18-month group was significantly different from the curve of BW change of age-matched controls throughout the 14 days of observation (*p* = 0.035) (Supplementary Figure S2 and Table S7).

### 2.2. Thermoregulatory Effects of Urocortin 2 Injections

With regard to the change in core temperature (ΔTc) following the ICV injection of Ucn2 during the tested 14-day period, the multivariate test of the three-way repeated-measures ANOVA showed that, in addition to the time (F(17, 52) = 11.736, *p* < 0.0001) and the treatment (F(17, 52) = 9.40, *p* < 0.0001), the age factor (F(51, 155.618) = 1.943, *p* = 0.001) affected the acute rise in core temperature. The effect size was large in all cases. There was also a significant interaction between the treatment and the age (time * treatment * age: F(51, 155.618) = 1.497, *p* = 0.032).

With regard to the within-subject effects, the Greenhouse–Geisser correction was applied. The effects of time and treatment were significant (SS = 36.893, F(2.311, 157.119) = 57.329, *p* < 0.0001 for time and SS = 32.611, F(2.311, 157.19) = 50.675, *p* < 0.0001 for treatment). The effect size was strong for time and treatment. With regard to age, the effect was not significant (SS = 3.124, F(6.932, 157.119) = 1.618, *p* = 0.135 for age). There was a significant interaction between time, treatment and age (time * treatment * age: SS = 6.688, F(6.932, 157.119) = 3.464, *p* = 0.002). 

**Figure 3:**
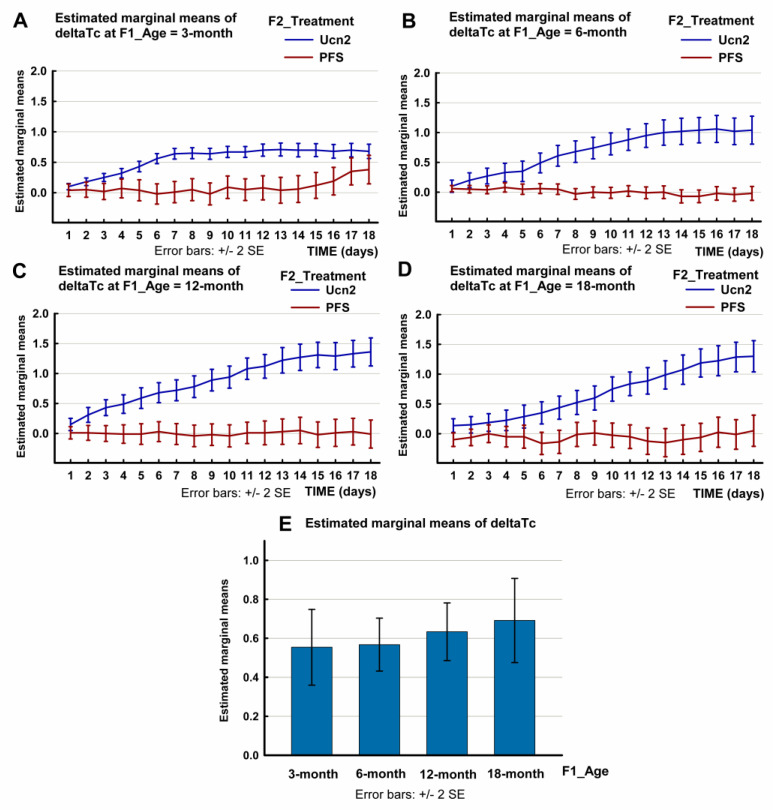
Changes in core temperature (ΔTc) of male Wistar rats of different age-groups (aged 3, 6, 12 or 18 months) following an intracerebroventricular (ICV) injection of urocortin 2 (Ucn2) or pyrogen-free saline (PFS). The plots show the estimated marginal mean values of the ΔTc of Ucn2-treated and PFS-treated control rats. (SPSS repeated-measures ANOVA, Profile Plots: change in core temperature; time * F2_Treatment * F1_Age). The bar chart (below) shows the estimated marginal mean 14-day ΔTc values of Ucn2-treated animals of various age groups. Data are also shown in Supplementary Tables S3 and S5. Number of animals per group: 3-month-old treated n = 10, 3-month-old control n = 10, 6-month-old treated n = 10, 6-month-old control n = 10, 12-month-old treated n = 10, 12-month-old control n = 10, 18-month-old treated n = 8, 18-month-old control n = 8).

Between-subject effects were significant only for treatment (SS = 164.774, F(1, 68) = 183.195, *p* < 0.0001). There was a significant interaction between treatment and age (treatment * age SS = 8.184, F(3, 68) = 3.033, *p* = 0.035. The plots showing the estimated marginal mean values are shown in Figure 3 and in Supplementary Tables S3 and S5.

We also compared the ΔTc of the Ucn2-treated rats with that of control animals for all the groups and within each age group. This analysis also showed that the increase in Tc varied with aging during the tested 14-day period. (Supplementary Figure S3 and Table S8). In the 3-month-old rats, the maximal rise in Tc reached 0.71 ± 0.12 °C at 130 min post-injection (repeated measures ANOVA, F(1, 14) = 25.324, *p* < 0.0001). In the 6-month-old animals, the maximal increase in Tc reached 0.83 ± 0.09 °C (F(1, 35) = 69.785, *p* < 0.0001); the 12-month-old group showed an even bigger hyperthermic response of 1.01 ± 0.13 °C (F(1, 31) = 33.692, *p* < 0.0001), exceeded only by the oldest 18-month-old animals, with a maximal change in Tc of 1.30 ± 0.18 °C (F(1, 12) = 26.61, *p* < 0.0001). There is a significant difference between the hyperthermic responses of the youngest 3- and 6-month-old and the oldest groups. The maximal Ucn2-induced rise in Tc was significantly higher in the oldest rats as compared with the 3-month (*p* = 0.011) and with the 6-month rats (*p* = 0.027). 

### 2.3. Effects of Urocortin 2 Injections on Oxygen Consumption

Regarding the change in oxygen consumption (ΔVO_2_) following the ICV injection of Ucn2 during the tested 14-day period, the multivariate test of the three-way repeated-measures ANOVA showed that time did not have a significant effect on the acute changes in oxygen consumption (F(17, 52) = 0.870, *p* = 0.609). On the other hand, the treatment (F(17, 52) = 2.045, *p* = 0.025) and the age factor (F(51, 155.618) = 1.963, *p* = 0.001) affected the acute changes in oxygen uptake. The effect size was large in both cases. There was no significant interaction between the treatment and the age (time * treatment * age: F(51, 155.618) = 1.220, *p* = 0.178.

With regard to the within-subject effects, the Greenhouse–Geisser correction was applied. The effects of treatment and age were significant (SS = 106.027, F(5.433, 369.437) = 4.798, *p* < 0.0001 for treatment and SS = 452.301, F(16.299, 369.437) = 6.822, *p* < 0.0001 for age). The effect size was medium for treatment and large for age. With regard to time, the effect was not significant (SS = 24.306, F(5.433, 369.437) = 1.100, *p* = 0.361). However, there was a significant interaction between time, treatment and age (time * treatment * age: SS = 168.567, F(16.299, 369.437) = 2.543, *p* = 0.001). 

**Figure 4:**
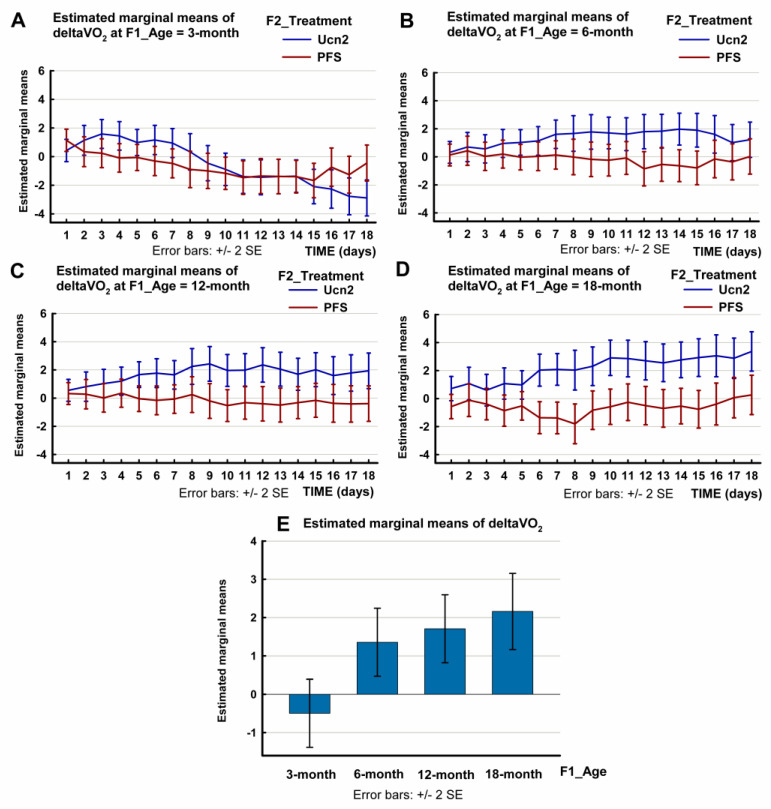
Changes in oxygen consumption (ΔVO_2_) of male Wistar rats of different age-groups (aged 3, 6, 12 or 18 months) following an intracerebroventricular (ICV) injection of urocortin 2 (Ucn2) or pyrogen-free saline (PFS). The plots show the estimated marginal mean values of the ΔVO_2_ of Ucn2-treated and PFS-treated control rats. (SPSS repeated-measures ANOVA, Profile Plots: change in oxygen consumption; time * F2_Treatment * F1_Age). The bar chart (below) shows the estimated marginal mean 14-day ΔVO_2_ values of Ucn2-treated animals of various age groups. Data are also shown in Supplementary Tables S4 and S5. Number of animals per group: 3-month-old treated n = 10, 3-month-old control n = 10, 6-month-old treated n = 10, 6-month-old control n = 10, 12-month-old treated n = 10, 12-month-old control n = 10, 18-month-old treated n = 8, 18-month-old control n = 8).

Between-subject effects were significant for treatment (SS = 847.450, F(1, 68) = 23.845, *p* < 0.0001) and age (SS = 450.296, F(3, 68) = 4.223, *p* = 0.008). There was no significant interaction between treatment and age (treatment * age SS = 289.364, F(3, 68) = 2.714, *p* = 0.052. The plots showing the estimated marginal mean values are shown in Figure 4 and in Supplementary Tables S4 and S5.

We also compared the ΔVO_2_ of the Ucn2-treated rats with that of control animals for all the groups and within each age group. This analysis also showed that the increase in VO_2_ varied with aging during the tested 14-day period. Upon the administration of the peptide, the VO_2_ increased for 180 min in most of the age groups (as compared to their age-matched controls) except in young adult rats (repeated measures ANOVA, F(7, 56) = 4.934, *p* < 0.0001) (Supplementary Figure S3 and Table S9). 

In the young adult 3-month-old rats, the rise in VO_2_ was observed only during the first 30 min post injection (repeated measures ANOVA, F(1, 10) = 5.996, *p* = 0.034). In the 6-month-old animals, the increase in VO_2_ reached 1.98 ± 0.73 mL/kg/min (F(1, 13) = 8.32, *p* = 0.013); the 12-month-old group showed and even bigger hypermetabolic response of 2.43 ± 0.88 mL/kg/min (F(1, 17) = 7.064, *p* = 0.017), exceeded only by the oldest 18-month-old animals, with a maximal change in VO_2_ of 3.45 ± 0.49 mL/kg/min (F(1, 12) = 28.30, *p* < 0.0001). There is a significant difference between the hypermetabolic responses of the youngest 3-month-old and the oldest groups. The maximal Ucn2-induced rise in VO_2_ was significantly higher in the oldest rats as compared with the 3-month-old animals (independent samples *t* test: *p* = 0.034).

### 2.4. Urocortin 2 mRNA Level in the Paraventricular Nucleus of the Hypothalamus (PVN)

The mRNA level of Ucn2 showed age-related changes in the PVN (one-way ANOVA F(3, 71) = 3.241, *p* = 0.027). The Ucn2 mRNA level decreased in the 12-month-old middle-aged group as compared with the 3-month-old group. (Tukey’s post hoc test *p* = 0.019). The mRNA level showed a tendency to increase again in the aging 18-month-old rats (Figure 4). 

## 3. Discussion

In our study, we aimed to investigate the age-related changes in the anorexigenic and hypermetabolic/hyperthermic responsiveness to centrally injected Ucn2 in male Wistar rats. 

It is well-known that centrally applied Ucn2 elicits anorexia and hyperthermia [20,24,25,26] via activation of the central CRH type 2 receptors [21] We hypothesized that the responsiveness to Ucn2 changes during the course of aging, similarly to other catabolic mediators, such as leptin or alpha-MSH [6,7,27,28]. The responsiveness to these mediators decreased in the middle-aged and increased in the old-age groups of laboratory rodents. Such age-related regulatory changes would promote weight gain in the middle-aged and weigh loss in the old animals. However, no previous study described the age-related changes in the central responsiveness or hypothalamic expression of Ucn2 during aging. 

**Figure 5:**
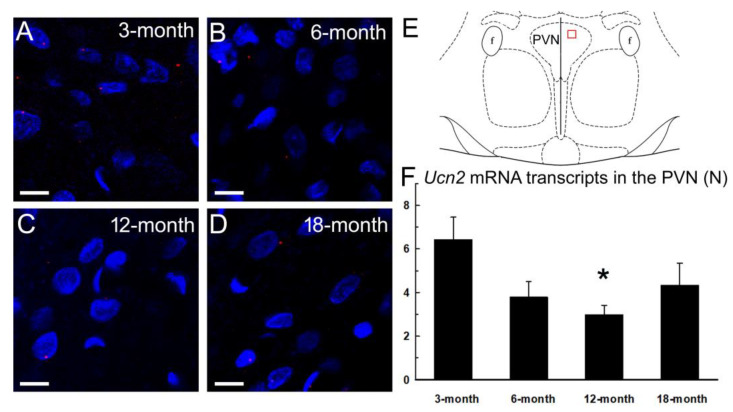
Age-dependent urocortin 2 (Ucn2) mRNA expression in the paraventricular nucleus of the hypothalamus (PVN). Representative digital photomicrographs show Ucn2 (red) mRNA transcripts in in 3- (**A**), 6- (**B**), 12- (**C**) and 18-month-old (**D**) rats (n = 5/group). Blue nuclear counterstaining with 4′,6-diamidino-2-phenylindole (DAPI) was applied. The images illustrate the red boxed PVN area shown in scheme (**E**) (Bregma −1.56 mm, for more information see Paxinos and Watson, 2007 [29]). Histogram (**F**) shows the number (N) of Ucn2 mRNA transcripts counted in the marked PVN areas. * *p* < 0.05, according to one-way ANOVA with Tukey’s post hoc test, compared to the 3-month-old group. f: fornix. Bars: 10 µm.

With regard to the anorexigenic effects, an acute ICV injection of Ucn2 reduced food intake in the 3-, 6- and 18-month-old groups, but failed to induce anorexia in the middle-aged 12-month-old animals (Figure 1, Supplementary Figure S1). Ucn2-induced anorexia was long-lasting; it remained significant for 7 days in the young adult 3-month-old group, for 14 days in the 6-month-old younger middle-aged group, and for 2 days in the oldest 18-month-old group. The fact that the older middle-aged (12-month) rats failed to show anorexia indicates a possible role of Ucn2 in the development of middle-aged obesity. The reduced expression of Ucn2 in the PVN of older middle-aged rats (significant difference between the 3- and the 12-month groups) also supports this hypothesis (Figure 5).

We also tested the acute hypermetabolic, hyperthermic effects of centrally applied Ucn2. Mediators that decrease body weight, such as leptin or melanocortin agonist alpha-melanocyte-stimulating hormone, not only show anorexigenic effects but also show hypermetabolic effects (as indicated by the increase in oxygen consumption) [6,28]. Such hypermetabolic effects also increase the core temperature. Such an increase in energy consumption acts synergistically with the decrease in FI. Although we recorded the acute hypermetabolic, hyperthermic responses to Ucn2 for 180 min, this provides an indication of how the hypermetabolic efficacy of Ucn2 changes during aging.

A hyperthermic response following ICV Ucn2 injection was observable in all age groups. These responses increased with aging (Figure 3, Supplementary Figure S3). The maximal rise in Tc was biggest in the 18-month-old rats and significantly higher than in the 3-month-old and 6-month-old rodents. Along with these hyperthermic reactions, the hypermetabolic effects of Ucn2 (indicated by the rise in oxygen consumption) increased for 180 min in most of the age groups (as compared to their age-matched controls), except in the youngest rats. In these young adult rats, the oxygen consumption increased only for 30 min. A rearrangement of the ratios of mitochondrial ATP synthesis and uncoupling protein-associated heat production (an estimated 80% and 20% under basal conditions, respectively) may be hypothesized as the cause of the hyperthermic response that occured without a strong and long-lasting increase in oxygen consumption [30]. The hypermetabolic effects increased with aging, with a significant difference between the youngest and oldest age groups (Figure 4, Supplementary Figure S3). 

Based on our results, it appears that the anorexigenic and hypermetabolic effects of Ucn2 show disparate age-related changes; that is, they change differently during aging. While the anorexigenic effects are strong in the younger middle-aged (6-month) group, they decline in the older middle-aged (12-month) animals and increase again, albeit slightly, in aging (18-month) rats. In contrast, the hypermetabolic responsiveness increases with aging, becoming especially strong in the oldest animals. 

It seems that anorexia predominantly determines weight loss, since 12-month-old rats that showed a strong hypermetabolic response to Ucn2, but no anorexia, failed to lose weight. However, the 18-month-old rats, in which a 2-day anorexic response was observed following a single central Ucn2 administration, showed a strong hypermetabolic/hyperthermic response. The BW of these rats decreased by a small but significant extent and remained lower throughout the observation period (Figure 2, Supplementary Figure S2). These findings also support the hypothesis that the decrease in FI is the major factor in weight loss. Nevertheless, the strong hypermetabolic response may help to maintain the achieved weight loss.

Our findings may also indicate that the anorexigenic and hypermetabolic responsiveness to Ucn2 depends on the activation of neurons in different hypothalamic nuclei. The age-related changes in the expression of Ucn2 mRNA in the PVN show a correlation with the changes in anorexigenic responsiveness, but no correlation with the changes in hypermetabolic responsiveness. The hypermetabolic effects of Ucn2 may depend more on the medial preoptic area of the hypothalamus, where the thermoregulatory center is found [31]. 

Our experimental findings seem to confirm, in part, our hypothesis. The age-dependent changes in the anorexigenic efficacy of Ucn2 are remarkably similar to those of melanocortin agonist alpha-MSH and also resemble those of leptin. The age-related pattern of hypermetabolic responsiveness to Ucn2 seems to be unique, since it increases with aging. In contrast, the hypermetabolic/hyperthermic responsiveness to leptin decreases with aging [6], and those of alpha-MSH show a U-shaped curve that reaches its nadir at the age of 12 months and then increases once again in the aging and old rats [28]. 

Concerning the sum of central Ucn2 effects, body weight decreased following the single central injection of Ucn2 in the 3-, 6- and 18-month age groups (Figure 2, Supplementary Figure S2). This weight loss was long-lasting. The body-weight change remained significantly different from that of age-matched controls for 10 days in the 3-month and for 14 days in the 6- and 18-month groups. Older middle-aged (12-month) rats failed to lose weight following the central Ucn2 injection. These results showed a similar pattern to the age-related changes in Ucn2 mRNA expression in the PVN (Figure 5).

Our observations with regard to age-dependent changes in Ucn2-induced anorexia and weight loss are in accordance with the literature data that show a complex relationship between hypothalamic anorexigenic mediators. It is well-known that leptin activates alpha-MSH release [32]. With regard to the relationship between leptin and Ucn2, earlier in vitro studies using hypothalamic cell lines demonstrated that leptin can increase the expression and activation of Ucn 2 and other members of the corticotropin peptide family (urocortin 3) in the hypothalamus [10,33]. Other researchers reported deficient leptin responsiveness in CRH2R-deficient mice [34]. 

Our study is the first to focus on the age-related changes in the anorexigenic and hypermetabolic responsiveness to centrally applied Ucn2. This mediator has advantageous characteristics as an anorexigenic substance, since a single injection induces an at least 14-day reduction in FI in the most sensitive younger middle-aged group. Therefore, daily application is unecessary. Our study also highlighted the best target population, i.e., younger middle-aged adults, in whom this peptide may contribute to the prevention of middle-aged obesity without malaise [35]. Thus, this mediator may be a worthy target of future research.

As a limitation of our study, we have to emphasize that we reported observations of animal experiments. No algorithm to assess odds ratios of obesity was applied. Our results suggest that Ucn2 would not be an effective treatment for middle-aged obesity, since it fails to induce anorexia or weight loss in the older middle-aged group. However, it may become useful in the prevention of middle-aged obesity if applied in younger middle-aged populations that show strong responsiveness to this mediator. 

However, Ucn2 may also be associated with side effects. Based on earlier clinical studies, intravenous Ucn2 infusions decrease blood pressure due to vasodilation. On the one hand, this may be a useful effect in cases of hypertension, along with the other beneficial effects of the peptide on the myocardium, as demonstrated in animals and in vitro studies. On the other hand, the frequent sinus tachycardia or the rarely developing ventricular tachycardia elicited by the Ucn2 treatment may present a remarkable cardiovascular complication [36,37]. 

In older age groups, the increasing hypermetabolic responses and the somewhat improved anorexigenic effects may help with weight loss in overweight or obese elderly individuals. However, the progressive loss of active tissues in the elderly would promote sarcopenic obesity in these high-risk populations, leading to frailty. Thus, in older groups, the cost–benefit ratio would very likely prove to be disadvantageous.

## 4. Materials and Methods

### 4.1. Animals

Different age groups of male Wistar rats from the Colony of the Institute for Translational Medicine of the Medical School, University of Pécs, Hungary were used in the present study. At the age of 3 (young adult), 6 (younger middle-aged), 12 (older middle-aged) and 18 (aging) months, they were placed individually into plastic cages (37.5 cm × 21.5 cm × 18 cm) covered with steel grids. They were fed a standard laboratory rat chow (11 kJ/g; CRLT/N rodent chow, Szindbád Kft., Gödöllő, Hungary). Food and water were available ad libitum. Animals were housed under a 12 h/12 h dark/light cycle and under an ambient temperature range of 22–25 °C. All animals were accustomed to regular handling and to the experimental conditions prior to the tests.

### 4.2. Surgical Intervention

Intracerebroventricular (ICV) 22-gauge stainless-steel guide cannula were implanted into the right lateral cerebral ventricle of the rats for the ICV injections. General anesthesia was performed during the surgery with an intraperitoneally (IP) injected combination of ketamine and xylazine (78 mg/kg (Calypsol, Richter) + 13 mg/kg (Sedaxylan, Eurovet)). A total of 2 mg IP Gentamycin was also administered to prevent infections. During surgery, first the head of the rats were fixed into the stereotaxic apparatus, then the skin was incised over the skull and the bone was cleaned. After these steps, two holes were drilled for miniature screws, and another one for the guide cannula. The placement of the guide cannula was determined by the Rat Brain Atlas of Paxinos and Watson [29]. The parameters were as follows: A: −1.0 mm to the bregma, L: 1.5 mm right lateral to the bregma and V: 3.8 mm ventral to the dura. The screws and the cannula were fixed with dental cement to the skull. A stylet closed the guide cannula, which was replaced during the experiments with a 28-gauge injection cannula, outreaching the guide cannula by about 0.5 mm [6].

The appropriate placement of the guide cannula was checked by the ICV administration of prostaglandin E_2_ (Sigma-Aldrich, Budapest, Hungary, P5515, 500 ng/5 μL). This substance was injected through a pp10 polyethylene (Portex) tube attachment 2 days before the tests. Appropriate location was confirmed if an at least 1.0 °C rise in Tc occurred within 60 min following the ICV injection. After the experiments, rats were euthanized by an overdose of an IP injection of urethane (3–5 g/kg, Reanal, Budapest, Hungary).

### 4.3. Post Mortem

We checked the injection sites macroscopically by coronal sections of the removed and fixed brains. Only those rats with appropriate cannula location were included in the statistical analysis [6].

### 4.4. Substances Applied

Ucn2 was obtained from Bachem (Switzerland, Product-No. 4040984). The peptide was dissolved in pyrogen-free saline (PFS) in 1 μg/μL or 0.2 μg/μL concentrations. For FI measurements, 1 μg Ucn2 was administered in a 5 μL volume; for the thermoregulatory tests, 5 μg Ucn2 was given in a similar 5 μL volume. Control animals received 5 μL PFS alone.

### 4.5. Assessment of the Food Intake

The recording of the consumed food was carried out in an automated FeedScale system (Columbus, OH, USA). Rats used in the analysis of FI) were given a powdered form of chow for at least 10–14 days before (habituation) and also during the experiments in order to avoid hoarding behavior. The different animal age groups (3-, 6-, 12-, 18-month-old) were transferred to the system 10–14 days before the experiments to become habituated to the environment. The amount of consumed food was recorded by the system every 30 min. Every 24 h, the system was restarted to record 24 h cumulative FI. The ICV injections (Ucn2 or control PFS) were given 5 min before starting the recording on the first day at 06:00 p.m. After the ICV injections, the follow-up period lasted for 14 days. Body weights were measured manually daily before 6 p.m.

### 4.6. Assessment of Thermoregulatory Functions

Thermoregulatory tests were performed in an indirect calorimeter system (Oxymax, Equal Flow, Columbus, OH, USA). The tests were performed on partially restrained rats, singly enclosed in cylindrical wire-mesh confiners in separated metabolic chambers (size: 20 × 30 × 18.5 cm). Previously, the animals were accustomed to the cages for at least a week so that we could minimize stress during the experiments [6]. The core temperature (Tc), the tail-skin temperature (Ts, indicating heat loss) and the ambient temperature (Ta) were measured with thermocouples attached to a Benchtop thermometer (Cole-Parmer). Temperature data were recorded on the computer. During the experiments, oxygen consumption (VO_2_, representing metabolic rate) was also detected every 10 min. With regard to heat loss, heat loss index (HLI) was calculated as follows: (Ts − Ta)/(Tc − Ta). The value of HLI changes from zero (indicating maximal vasoconstriction, i.e., Ts equals Ta) to one (indicating maximal skin vasodilation, i.e., Ts equals Tc). 

### 4.7. Investigation of Age-Related Changes in the Ucn2 mRNA Expression in the PVN of the Hypothalamus Using RNAscope

Rats were intraperitoneally anesthetized with an overdose of urethane (2.8 g/kg, Merck KGaA, Darmstadt, Germany) and transcardially perfused with 50 mL 0.1 M phosphate-buffered saline (PBS, pH 7.4), followed by 250 mL 4% paraformaldehyde in Millonig’s buffer. Brains were dissected and post-fixed.

A total of 30 μm coronal vibratome (Leica Biosystems, Wetzlar, Germany) sections between −1.5 mm and −2.00 mm to the bregma (Paxinos and Watson, 2007), including the paraventricular nucleus of the hypothalamus (PVN), were collected and stored in PBS containing 0.01% sodium azide at 4 °C. Two coronal sections per animal, interspaced by 150 µm, were selected, which bilaterally contained the PVN. The sections were subjected to a modified pretreatment procedure for RNAscope, as recently published by us [38,39]. Subsequent steps of the RNAscope protocol were performed according to the supplier’s suggestions. The urocortin 2 (Ucn2) mRNA was visualized by Cy3 (1:3000) using a rat Ucn2 probe (Cat No: 829641-C2, Advanced Cell Diagnostics, Newark, CA, USA). Sections were counterstained with 4′,6-diamidino-2-phenylindole (DAPI) and covered with antifade medium.

Triplex positive (Cat No: 320891) and negative control (Cat No: 320871) probes were used to prove the sensitivity of the test on randomly selected sections. The positive control gave an obviously recognizable signal, while no fluorescence was seen in the negative control (images not shown).

### 4.8. Microscopy, Digitalization and Morphometry

Four PVN cross-section areas per animal were digitalized using a Olympus FluoView 1000 confocal microscope with ×40 (NA:0.8) objectives in analog mode. The excitation and emission of fluorophores were set according to the built-in settings of the FluoView software (Fv10-ASW; Version 0102). Blue (DAPI) and red (Cy3) virtual colors were assigned to the dyes.

Signal dots of the mRNA labeling were manually counted on four non-edited digital images per animal by two independent researchers (DKK and ESZ) using the ImageJ software (version 1.52a, NIH). The average of the four cross-section surface areas was averaged, and this number represented one animal in the statistical assessment. For publication, selected representative images were cropped, contrasted and edited using Adobe Photoshop software.

### 4.9. Statistical Analysis

For the statistical analysis of our data, the SPSS for Windows 25.0 software was used. A three-way repeated-measures ANOVA was carried out, in which one within-subject factor (time) and two between-subject factors (treatment and age) were applied for the analysis of FI, ΔBW, ΔTc and ΔVO_2_. We included a description of the results of the multivariate tests (the Wilks’ Lambda tests), and the results of tests of the within-subjects effects to which we needed to apply the Greenhouse–Geisser correction of the degrees of freedom values (df-s) in all cases (based on the significant results of the Mauchly's test of sphericity and on the epsilon values). For the sums of squares, the default Type III was used. In addition, we described the results of the tests of between-subjects effects. The plots of the estimated marginal means (created by the SPSS for Windows 25.0 software) were used to demonstrate our results (Figure 1, Figure 2, Figure 3 and Figure 4). These data are provided in the Supplementary Tables (Supplementary Tables S1–S4). Data of additional analyses (repeated-measures ANOVA) comparing the results of the Ucn2-treated and the control groups within each age group are also provided in the Supplementary Material. Age-related changes in the Ucn2 mRNA expression in the PVN of the hypothalamus was analysed by one-way ANOVA with Tukey’s post hoc test (Figure 5). The significance was set at the level of *p* < 0.05. Figure 5 and Supplementary Figures S1–S3 were prepared with the SigmaPlot 11.0 software. These latter results are shown as mean ± SEM.

## 5. Conclusions

The sum of the central effects of Ucn2 on energy metabolism changes with aging way suggest a role for this peptide in the development of both middle-aged obesity and aging anorexia and hypermetabolism leading to cachexia. In the future, this peptide or its analogues may be best applied in the prevention and/or treatment of obesity in the younger middle-aged populations.

## Data Availability

The data presented in this study are available on request from the corresponding author.

## Supplementary Materials

The following supporting information can be downloaded at: https://www.mdpi.com/article/10.3390/ijms24108992/s1.
