# Supplementary material for: Aging Changes the Efficacy of Central Urocortin 2 to Induce Weight Loss in Rats
Source: Int J Mol Sci. 2023 May 19;24(10):8992. doi: 10.3390/ijms24108992 (PMC10219457; doi:10.3390/ijms24108992)
Supplement: Supplementary file 1 [file ijms-24-08992-s001.zip › ijms-2350217-supplementary.pdf]

International Journal of *Molecular Sciences*

MDPI

Article

Aging Changes the Efficacy of Central Urocortin 2 to Induce Weight Loss in Rats

Dóra K. Kovács [1], Szimonetta Eitmann [1], Gergely Berta [2], Viktória Kormos [3], Balázs Gaszner [4], Erika Pétervári [1] and Márta Balaskó [1,*]

[1] Institute for Translational Medicine, Medical School, University of Pécs, Szigeti út 12., 7624, Pecs, Hungary
[2] Department of Medical Biology, Medical School, University of Pécs, Szigeti út 12., 7624, Pecs, Hungary
[3] Department of Pharmacology and Pharmacotherapy, Medical School, University of Pécs, Szigeti út 12., 7624, Pecs, Hungary
[4] Department of Anatomy, Medical School, University of Pécs, Szigeti út 12., 7624, Pecs, Hungary
* Correspondence: marta.balasko@aok.pte.hu

Supplementary Table S1 Estimated marginal means (± standard error of means) of daily food intake values (g) following a single intracerebroventricular injection of urocortin 2 or pyrogen-free saline (control) in different age-groups of male Wistar rats

Time (days)	3-month control	3-month Ucn2	6-month control	6-month Ucn2	12-month control	12-month Ucn2	18-month control	18-month Ucn2
**1**	26.36 ± 0.84	15.28 ± 1.39	26.37 ± 1.30	14.24 ± 1.16	24.00 ± 0.92	19.33 ± 1.50	22.33 ± 1.50	15.93 ± 1.30
**2**	24.84 ± 0.93	20.85 ± 1.54	24.25 ± 1.44	18.25 ± 1.29	24.06 ± 1.02	29.00 ± 1.67	05.22 ± 1.67	20.41 ± 1.44
**3**	24.42 ± 0.96	23.57 ± 1.58	22.87 ± 1.48	19.79 ± 1.32	25.56 ± 1.04	20.66 ± 1.70	23.00 ± 1.70	21.78 ± 1.48
**4**	24.00 ± 0.77	23.00 ± 1.27	22.87 ± 1.19	02.20 ± 1.06	24.12 ± 0.84	20.33 ± 1.37	21.00 ± 1.37	01.21 ± 1.19
**5**	24.21 ± 0.72	21.14 ± 1.19	24.12 ± 1.11	20.27 ± 0.99	23.75 ± 0.78	21.00 ± 1.28	19.66 ± 1.28	19.81 ± 1.11
**6**	24.10 ± 0.67	23.14 ± 1.10	24.00 ± 1.03	21.41 ± .092	23.06 ± 0.73	21.00 ± 1.19	19.83 ± 1.19	19.26 ± 1.03
**7**	25.42 ± 0.68	22.85 ± 1.13	25.00 ± 1.06	09.20 ± 0.94	24.25 ± 0.75	19.33 ± 1.22	23.83 ± 1.22	20.12 ± 1.06
**8**	25.47 ± 0.81	24.85 ± 1.34	25.12 ± 1.26	20.37 ± 1.12	25.43 ± 0.89	20.33 ± 1.45	22.16 ± 1.45	22.45 ± 1.26
**9**	25.73 ± 0.78	23.85 ± 1.28	05.25 ± 1.20	20.92 ± 1.07	25.12 ± 0.84	23.00 ± 1.38	22.00 ± 1.38	22.98 ± 1.20
**10**	23.78 ± 0.78	26.42 ± 1.29	05.22 ± 1.21	04.21 ± 1.08	25.93 ± 0.85	19.33 ± 1.40	22.00 ± 1.40	22.58 ± 1.21
**11**	23.36 ± 0.73	24.28 ± 1.20	21.87 ± 1.12	20.72 ± 1.00	25.00 ± 0.79	21.33 ± 1.30	22.33 ± 1.30	20.58 ± 1.12
**12**	24.10 ± 0.79	23.42 ± 1.31	23.87 ± 1.22	20.49 ± 1.09	05.23 ± 0.86	22.33 ± 1.41	22.66 ± 1.41	05.23 ± 1.22
**13**	24.36 ± 0.74	22.42 ± 1.22	25.12 ± 1.14	21.29 ± 1.02	25.31 ± 0.81	21.33 ± 1.32	23.66 ± 1.32	21.96 ± 1.14
**14**	23.36 ± 0.84	25.57 ± 1.39	05.24 ± 1.30	04.20 ± 1.17	24.62 ± 0.92	20.33 ± 1.51	22.16 ± 1.51	21.51 ± 1.30

Supplementary Table S2 Estimated marginal means (± standard error of means) of the changes in body weight (g) following a single intracerebroventricular injection of urocortin 2 or pyrogen-free saline (control) in different age-groups of male Wistar rats

Time (days)	3-month control	3-month Ucn2	6-month control	6-month Ucn2	12-month control	12-month Ucn2	18-month control	18-month Ucn2
**1**	-1.84 ± 1.11	-8.14 ± 1.84	-1.00 ± 1.72	-12.60 ± 1.54	0.53 ± 1.35	-1.66 ± 1.99	3.20 ± 2.18	-7.90 ± 1.47
**2**	0.73 ± 1.22	-2.57 ± 2.00	1.37 ± 1.87	-11.50 ± 1.68	0.38 ± 1.47	1.66 ± 2.17	-0.40 ± 2.37	-3.90 ± 1.60
**3**	3.52 ± 1.24	0.14 ± 2.04	1.87 ± 1.91	-8.60 ± 1.71	1.69 ± 1.50	0.00 ± 2.20	-0.19 ± 2.41	-4.00 ± 1.63
**4**	4.00 ± 1.16	-0.42 ± 1.91	3.87 ± 1.79	-7.10 ± 1.60	-0.30 ± 1.40	0.33 ± 2.07	1.20 ± 2.26	-4.36 ± 1.52
**5**	6.00 ± 1.37	-2.28 ± 2.27	6.62 ± 2.12	-5.50 ± 1.90	0.61 ± 1.66	2.67 ± 2.45	1.80 ± 2.68	-6.09 ± 1.81
**6**	6.63 ± 1.59	-0.28 ± 2.62	5.12 ± 2.45	-5.80 ± 2.19	2.38 ± 1.92	-0.67 ± 2.83	1.60 ± 3.10	-6.63 ± 2.09
**7**	8.73 ± 1.52	-0.57 ± 2.50	7.25 ± 2.34	-5.60 ± 2.09	1.76 ± 1.83	-2.00 ± 2.70	0.00 ± 2.96	-4.36 ± 1.99
**8**	9.57 ± 1.37	1.42 ± 2.26	7.12 ± 2.11	-3.10 ± 1.89	2.00 ± 1.66	-1.67 ± 2.44	-0.99 ± 2.67	-4.72 ± 1.80
**9**	11.42 ± 1.28	0.42 ± 2.12	8.62 ± 1.98	-2.30 ± 1.77	1.30 ± 1.55	-1.33 ± 2.29	3.00 ± 2.50	-4.00 ± 1.69
**10**	12.47 ± 1.48	3.00 ± 2.44	9.12 ± 2.28	-2.20 ± 2.04	1.53 ± 1.79	-1.00 ± 2.63	4.20 ± 2.88	-4.18 ± 1.94
**11**	15.21 ± 1.65	0.85 ± 2.73	13.00 ± 2.55	-0.60 ± 2.28	2.46 ± 2.00	1.66 ± 2.95	1.20 ± 3.23	-5.18 ± 2.17
**12**	17.00 ± 1.64	0.00 ± 2.71	15.87 ± 2.53	-0.90 ± 2.26	2.92 ± 1.98	0.33 ± 2.92	4.00 ± 3.20	-3.18 ± 2.16
**13**	19.63 ± 2.02	-1.00 ± 3.32	16.50 ± 3.11	-0.10 ± 2.78	1.46 ± 2.44	-3.00 ± 3.59	2.79 ± 3.93	-6.00 ± 2.65
**14**	17.00 ± 2.23	2.14 ± 3.67	17.75 ± 3.43	3.80 ± 3.07	1.07 ± 2.69	0.66 ± 3.97	6.60 ± 4.35	-4.09 ± 2.93

Supplementary Table S3 Estimated marginal means (± standard error of means) of the changes in core temperature (°C) following a single intracerebroventricular injection of urocortin 2 or pyrogen-free saline (control) in different age-groups of male Wistar rats

Time (min)	3-month control	3-month Ucn2	6-month control	6-month Ucn2	12-month control	12-month Ucn2	18-month control	18-month Ucn2
**10**	0.04 ± 0.05	0.10 ± 0.05	0.06 ± 0.05	0.10 ± 0.05	0.01 ± 0.05	0.15 ± 0.05	-0.10 ± 0.05	0.13 ± 0.05
**20**	0.05 ± 0.06	0.18 ± 0.06	0.05 ± 0.06	0.20 ± 0.06	0.01 ± 0.06	0.31 ± 0.06	-0.06 ± 0.06	0.15 ± 0.06
**30**	0.02 ± 0.06	0.25 ± 0.06	0.04 ± 0.06	0.27 ± 0.06	0.00 ± 0.06	0.43 ± 0.06	0.00 ± 0.07	0.18 ± 0.07
**40**	0.07 ± 0.07	0.32 ± 0.07	0.08 ± 0.07	0.33 ± 0.07	-0.01 ± 0.07	0.49 ± 0.07	-0.05 ± 0.08	0.22 ± 0.08
**50**	0.04 ± 0.08	0.43 ± 0.08	0.05 ± 0.08	0.35 ± 0.08	-0.01 ± 0.08	0.59 ± 0.08	-0.05 ± 0.09	0.28 ± 0.09
**60**	-0.02 ± 0.08	0.56 ± 0.08	0.06 ± 0.08	0.49 ± 0.08	0.03 ± 0.08	0.68 ± 0.08	-0.16 ± 0.09	0.35 ± 0.09
**70**	0.01 ± 0.08	0.64 ± 0.08	0.05 ± 0.08	0.61 ± 0.08	-0.01 ± 0.08	0.72 ± 0.08	-0.13 ± 0.09	0.43 ± 0.09
**80**	0.05 ± 0.09	0.65 ± 0.09	-0.03 ± 0.09	0.68 ± 0.09	-0.04 ± 0.09	0.78 ± 0.09	-0.01 ± 0.10	0.52 ± 0.10
**90**	-0.02 ± 0.09	0.64 ± 0.09	-1.38 ± 0.09	0.74 ± 0.09	-0.02 ± 0.09	0.89 ± 0.09	0.01 ± 0.10	0.60 ± 0.10
**100**	0.09 ± 0.09	0.67 ± 0.09	-0.01 ± 0.09	0.81 ± 0.09	-0.04 ± 0.09	0.94 ± 0.09	-0.02 ± 0.10	0.75 ± 0.10

**110**	0.05 ± 0.08	0.67 ± 0.08	0.02 ± 0.08	0.88 ± 0.08	0.01 ± 0.08	1.08 ± 0.08	-0.05 ± 0.09	0.83 ± 0.09
**120**	0.08 ± 0.09	0.70 ± 0.09	-0.01 ± 0.09	0.95 ± 0.09	0.01 ± 0.09	1.12 ± 0.09	-0.12 ± 0.11	0.88 ± 0.11
**130**	0.04 ± 0.10	0.71 ± 0.10	2.49 ± 0.10	1.00 ± 0.10	0.03 ± 0.10	1.22 ± 0.10	-0.15 ± 0.11	0.98 ± 0.11
**140**	0.06 ± 0.11	0.70 ± 0.11	-0.07 ± 0.11	1.02 ± 0.11	0.05 ± 0.11	1.27 ± 0.11	-0.09 ± 0.12	1.07 ± 012
**150**	0.12 ± 0.10	0.70 ± 0.10	-0.07 ± 0.10	1.04 ± 0.10	-0.02 ± 0.10	1.31 ± 0.10	-0.06 ± 0.11	1.18 ± 0.11
**160**	0.19 ± 0.11	0.68 ± 0.11	-0.02 ± 0.11	1.06 ± 0.11	0.01 ± 0.11	1.29 ± 0.11	0.02 ± 0.12	1.22 ± 0.12
**170**	0.35 ± 0.11	0.70 ± 0.11	-0.04 ± 0.11	1.02 ± 0.11	0.03 ± 0.11	1.33 ± 0.11	-0.01 ± 0.12	1.28 ± 0.12
**180**	0.38 ± 0.11	0.68 ± 0.11	-0.02 ± 0.11	1.04 ± 0.11	-0.01 ± 0.11	1.36 ± 0.11	0.05 ± 0.13	1.30 ± 0.13

Supplementary Table S4 Estimated marginal means (± standard error of means) of the changes in oxygen consumption (ml/kg/min) following a single intracerebroventricular injection of urocortin 2 or pyrogen-free saline (control) in different age-groups of male Wistar rats

**Time (min)**	**3-month control**	**3-month Ucn2**	**6-month control**	**6-month Ucn2**	**12-month control**	**12-month Ucn2**	**18-month control**	**18-month Ucn2**
**10**	1.13 ± 0.38	0.42 ± 0.38	0.13 ± 0.38	0.32 ± 0.38	0.32 ± 0.38	0.55 ± 0.38	-0.56 ± 0.43	0.71 ± 0.43
**20**	0.34 ± 0.52	1.13 ± 0.52	0.43 ± 0.52	0.69 ± 0.52	0.27 ± 0.52	0.81 ± 0.52	-0.11 ± 0.58	1.07 ± 0.58
**30**	0.23 ± 0.50	1.58 ± 0.50	0.03 ± 0.50	0.56 ± 0.50	0.01 ± 0.50	1.03 ± 0.50	-0.39 ± 0.56	0.60 ± 0.56
**40**	-0.09 ± 0.49	1.44 ± 0.49	0.19 ± 0.49	0.95 ± 0.49	0.34 ± 0.49	1.20 ± 0.49	-0.86 ± 0.55	1.07 ± 0.55
**50**	-0.06 ± 0.45	0.98 ± 0.45	-0.02 ± 0.45	1.02 ± 0.45	-0.04 ± 0.45	1.66 ± 0.45	-0.52 ± 0.50	0.97 ± 0.50
**60**	-0.30 ± 0.51	1.16 ± 0.51	0.03 ± 0.51	1.13 ± 0.51	-0.16 ± 0.51	1.77 ± 0.51	-1.36 ± 0.57	2.03 ± 0.57
**70**	-0.47 ± 0.50	0.94 ± 0.50	0.13 ± 0.50	1.60 ± 0.50	-0.07 ± 0.50	1.64 ± 0.50	-1.38 ± 0.56	2.08 ± 0.56
**80**	-0.89 ± 0.63	0.33 ± 0.63	-0.01 ± 0.63	1.66 ± 0.63	0.25 ± 0.63	2.24 ± 0.63	-1.80 ± 0.70	2.03 ± 0.70
**90**	-0.99 ± 0.61	-0.45 ± 0.61	-0.18 ± 0.61	1.77 ± 0.61	-0.20 ± 0.61	2.42 ± 0.61	-0.82 ± 0.68	2.30 ± 0.68
**100**	-1,16 ± 0.56	-0.88 ± 0.56	-0.24 ± 0.56	1.70 ± 0.56	-0.53 ± 0.56	1.96 ± 0.56	-0.58 ± 0.63	2.90 ± 0.63
**110**	-1,46 ± 0.58	-1.39 ± 0.58	-0.08 ± 0.58	1.61 ± 0.58	-0.32 ± 0.58	1.98 ± 0.58	-0.26 ± 0.65	2.86 ± 0.65
**120**	-1,35 ± 0.61	-1.44 ± 0.61	-0.85 ± 0.61	1.79 ± 0.61	-0.40 ± 0.61	2.35 ± 0.61	-0.50 ± 0.68	2.69 ± 0.68
**130**	-1,38 ± 0.60	-1.39 ± 0.60	-0.54 ± 0.60	1.82 ± 0.60	-0.49 ± 0.60	2.05 ± 0.60	-0.69 ± 0.67	2.55 ± 0.67
**140**	-1,40 ± 0.56	-1.36 ± 0.56	-0.63 ± 0.56	1.97 ± 0.56	-0.32 ± 0.56	1.69 ± 0.56	-0.53 ± 0.63	2.76 ± 0.63
**150**	-1,66 ± 0.60	-2.09 ± 0.60	-0.79 ± 0.60	1.89 ± 0.60	-0.15 ± 0.60	2.01 ± 0.60	-0.76 ± 0.67	2.92 ± 0.67
**160**	-0,73 ± 0.67	-2.26 ± 0.66	-0.15 ± 0.66	1.60 ± 0.66	-0.37 ± 0.67	1.59 ± 0.67	-0.39 ± 0.74	3.05 ± 0.74

**170**	-1,26 ± 0.64	-2.77 ± 0.64	-0.36 ± 0.64	1.01 ± 0.64	-0.41 ± 0.64	1.78 ± 0.64	0.06 ± 0.72	2.87 ± 0.72
**180**	-0,44 ± 0.62	-2.88 ± 0.62	0.02 ± 0.62	1.21 ± 0.63	-0.38 ± 0.62	1.93 ± 0.63	0.26 ± 0.70	3.36 ± 0.70

Supplementary Table S5 Estimated marginal mean values for the whole 14-day observation period with regard to food intake (FI), body weight change (ΔBW), core temperature change (ΔTc) and change of oxygen consumption (ΔVO_2) of Ucn2-treated animals of various age-groups.

Parameters	3-month Ucn2	6-month Ucn2	12-month Ucn2	18-month Ucn2
**FI (g)**	22.91 ± 1.00	20.02 ± 0.84	21.33 ± 1.09	21.00 ± 0,9390
**ΔBW (g)**	-0.52 ± 1.88	-4.44 ± 1.57	-0.29 ± 2.03	-4.90 ± 1.50
**ΔTc (°C)**	0.55 ± 0.07	0.70 ± 0.07	0.89 ± 0.07	0.69 ± 0.08
**ΔVO_2 (ml/kg/min)**	-0.50 ± 0.44	1.36 ± 0.44	1.71 ± 0.44	2.16 ± 0.50

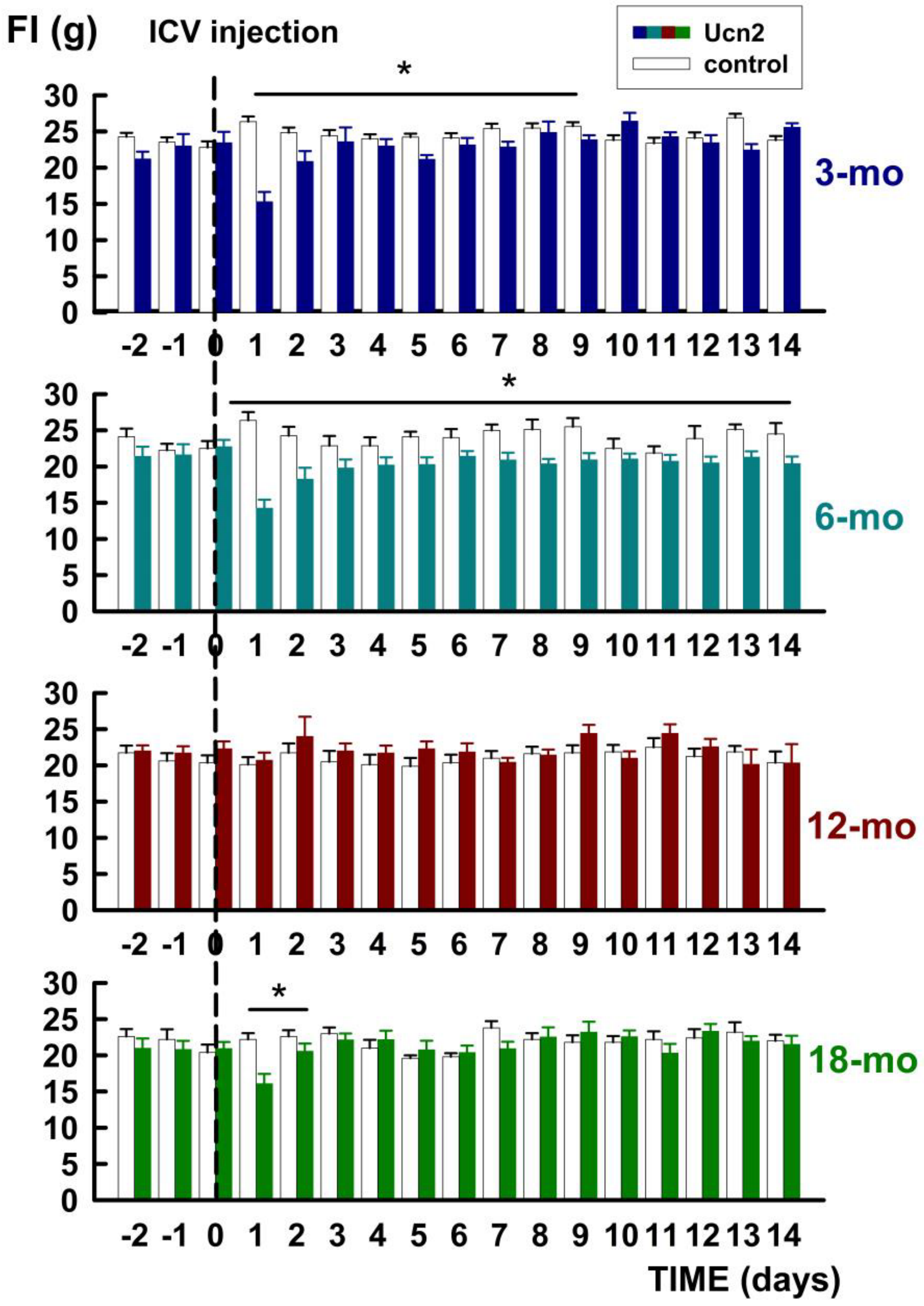

Supplementary Figure S1. Changes in daily food intake (FI) of male Wistar rats of different age-groups following an intracerebroventricular (ICV) injection of urocortin 2 (Ucn2). Dark coloums indicate Ucn2-treated, white coloums indicate results of age-matched controls. Control animals received pyrogen-free saline (PFS). Colours indicate age-groups (dark blue represents 3-month; dark cyan 6-month, dark red 12-month and dark green 18-month animals). Asterisks indicate significant differences between the Ucn2-treated and control animals shown by repeated-measures ANOVA. The length of the line shows the duration of the difference. Data (mean ± SEM) are also shown in Supplementary Table S1. Number of animals per group: 3-month treated n=7, 3-month control n= 8, 6-month treated n=10, 6-month control n= 8, 12-month treated n=6, 12-month control n= 8, 18-month treated n=8, 18-month control n= 6).

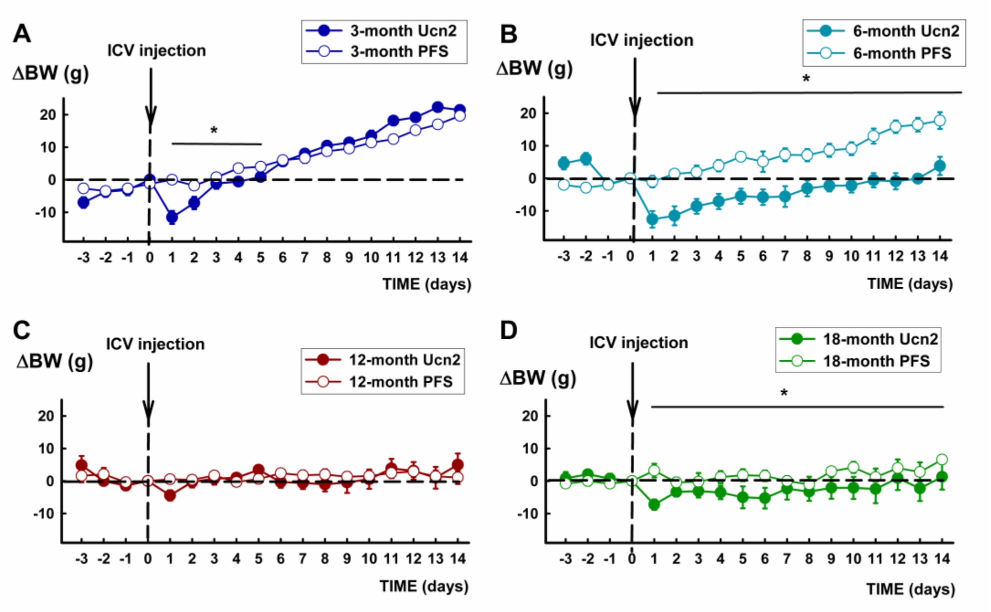

Supplementary Figure S2. Changes in body weight (ΔBW) of male Wistar rats of different age-groups following an intracerebroventricular (ICV) injection of urocortin 2 (Ucn2). Dark symbols indicate Ucn2-treated, empty symbols indicate results of age-matched controls. Control animals received pyrogen-free saline (PFS). Asterisks indicate significant differences between the Ucn2-treated and control animals shown by repeated-measures ANOVA. The length of the line shows the duration of the difference. Data (mean ± SEM) are also shown in Supplementary Table S2. Number of animals per group: 3-month treated n=7, 3-month control n= 8, 6-month treated n=10, 6-month control n= 8, 12-month treated n=6, 12-month control n= 8, 18-month treated n=8, 18-month control n= 6).

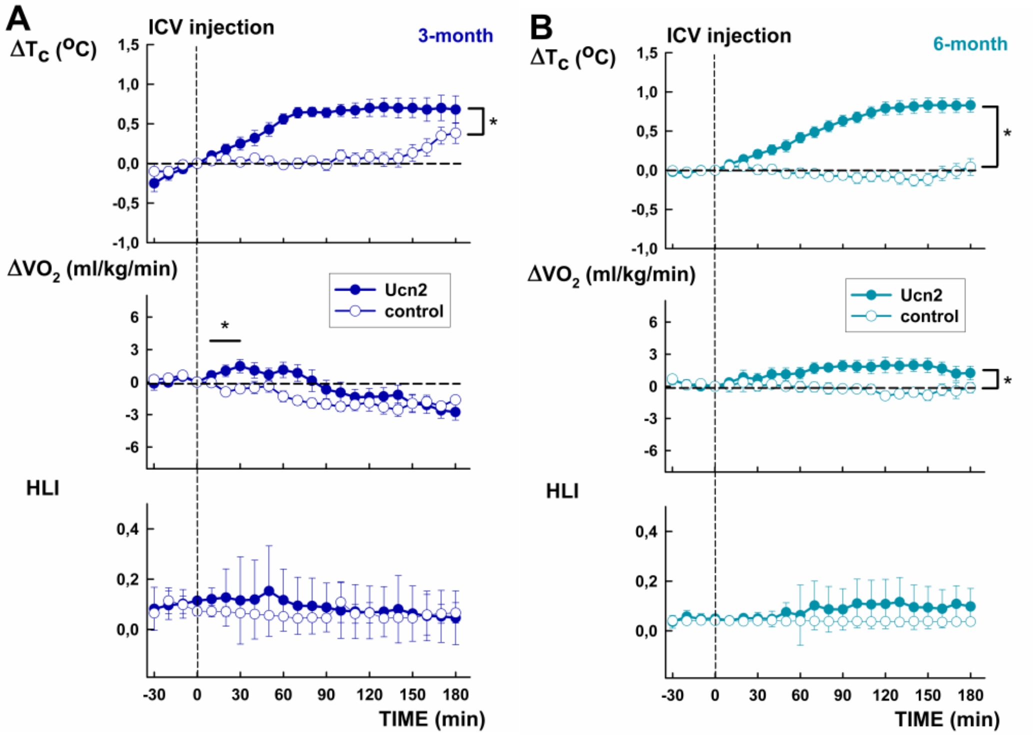

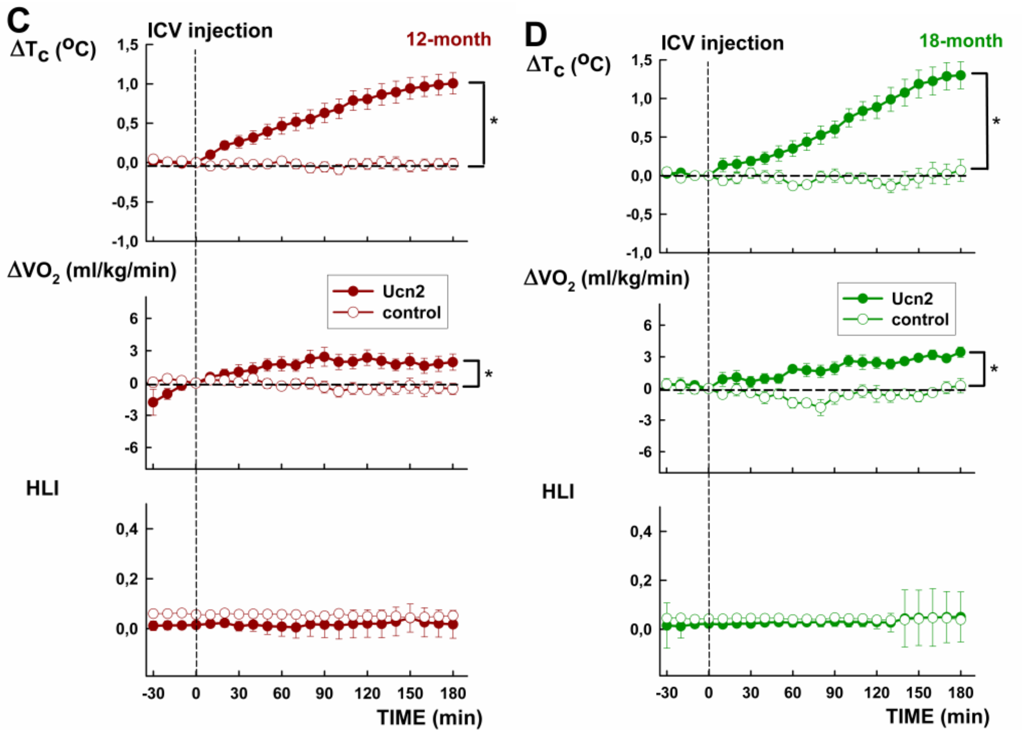

Supplementary Figure S3. Changes in core temperature (ΔTc), oxygen consumption (ΔVO_2) and heat loss index (HLI = (Ts-Ta)/(Tc-Ta)) of male Wistar rats of different age-groups following an intracerebroventricular (ICV) injection of urocortin 2 (Ucn2). Dark symbols indicate Ucn2-treated, empty symbols indicate results of age-matched controls. (Panels A, B, C and D shows the results obtained in 3-month, 6-month, 12-month and 18-month rats, respectively.) Control animals received pyrogen-free saline (PFS). Asterisks indicate significant differences between the Ucn2-treated and control animals shown by repeated-measures ANOVA. Ts: tail skin temperature; Ta: ambient temperature. Data (mean ± SEM) are also shown in Supplementary Tables S3-4. Number of animals per group: 3-month treated n=10, 3-month control n= 10, 6-month treated n=10, 6-month control n= 10, 12-month treated n=10, 12-month control n= 10, 18-month treated n=8, 18-month control n= 8).

Supplementary Table S6. Daily food intake before and after a single intracerebroventricular injection of urocortin 2

Time (days)	3-month control	3-month Ucn2	6-month control	6-month Ucn2	12-month control	12-month Ucn2	18-month control	18-month Ucn2
-2	24.26 ± 0.54	21.20 ± 1.01	24.12 ± 1.10	21.40 ± 1.34	21.75 ± 0.97	22.00 ± 0.75	22.60 ± 1.02	20.98 ± 1.35
-1	23.52 ± 0.65	23.00 ± 1.66	22.25 ± 0.88	21.60 ± 1.47	20.62 ± 1.05	21.71 ± 0.91	22.20 ± 1.39	20.80 ± 1.19
0	22.78 ± 0.84	23.42 ± 1.52	22.50 ± 1.01	22.73 ± 0.93	20.37 ± 1.01	22.28 ± 1.01	20.40 ± 1.07	20.93 ± 0.90
1	26.36 ± 0.69	15.28 ± 1.35	26.37 ± 1.14	14.24 ± 1.17	20.12 ± 1.00	20.71 ± 1.04	22.20 ± 0.86	16.08 ± 1.34
2	24.84 ± 0.70	20.85 ± 1.43	24.25 ± 1.23	18.25 ± 1.58	21.75 ± 1.27	24.00 ± 2.72	22.60 ± 0.87	20.55 ± 1.07
3	24.42 ± 0.77	23.57 ± 1.98	22.87 ± 1.34	19.79 ± 1.18	20.50 ± 1.50	22.00 ± 1.02	23.00 ± 0.83	22.14 ± 0.88
4	24.00 ± 0.60	23.00 ± 0.95	22.87 ± 1.15	20.20 ± 1.07	20.12 ± 1.35	21.71 ± 1.01	21.00 ± 1.14	22.16 ± 1.24
5	24.21 ± 0.49	21.14 ± 0.59	24.12 ± 0.69	20.27 ± 1.00	19.87 ± 1.15	22.28 ± 1.01	19.60 ± 0.40	20.76 ± 1.24
6	24.10 ± 0.66	23.14 ± 0.96	24.00 ± 1.16	21.41 ± 0.72	20.37 ± 1.10	21.85 ± 1.18	19.80 ± 0.48	20.39 ± 0.94

7	25.42 ± 0.65	22.85 ± 0.73	25.00 ± 0.80	20.90 ± 1.01	21.00 ± 0.98	20.42 ± 0.61	23.80 ± 0.91	20.91 ± 0.95
8	25.47 ± 0.64	24.85 ± 1.51	25.12 ± 1.36	20.37 ± 0.69	21.62 ± 0.94	21.42 ± 0.75	22.20 ± 0.86	22.53 ± 1.32
9	25.73 ± 0.54	23.85 ± 0.63	25.50 ± 1.19	20.92 ± 0.92	21.75 ± 1.03	24.42 ± 1.17	21.80 ± 0.96	23.20 ± 1.43
10	23.78 ± 0.69	26.42 ± 1.13	22.50 ± 1.34	21.04 ± 0.75	21.87 ± 0.95	21.00 ± 0.92	21.80 ± 0.86	22.57 ± 0.86
11	23.36 ± 0.77	24.28 ± 0.60	21.87 ± 0.91	20.72 ± 0.90	22.50 ± 1.26	24.42 ± 1.25	22.20 ± 1.11	20.30 ± 1.27
12	24.10 ± 0.76	23.42 ± 1.06	23.87 ± 1.72	20.49 ± 0.87	21.25 ± 1.06	22.57 ± 1.08	22.40 ± 1.20	23.32 ± 0.99
13	26.90 ± 0.54	22.42 ± 0.84	25.12 ± 0.69	21.29 ± 0.81	21.87 ± 0.81	20.14 ± 2.05	23.20 ± 1.35	21.96 ± 0.67
14	23.80 ± 0.57	25.57 ± 0.57	24.50 ± 1.50	20.40 ± 0.97	20.37 ± 1.54	20.33 ± 2.60	22.00 ± 0.83	21.51 ± 1.19

Supplementary Table S7. Changes in body weight before and after a single intracerebroventricular injection of urocortin 2

Time (days)	3-month control	3-month Ucn2	6-month control	6-month Ucn2	12-month control	12-month Ucn2	18-month control	18-month Ucn2
**-3**	-2.68 ± 0.65	-7.00 ± 1.64	-2.00 ± 1.17	4.60 ± 1.86	1.61 ± 2.00	4.83 ± 2.87	-0.80 ± 0.37	0.83 ± 1.96
**-2**	-3.47 ± 0.80	-3.71 ± 1.58	-2.87 ± 1.19	6.00 ± 1.82	2.07 ± 1.98	0.00 ± 1.31	0.00 ± 1.41	2.08 ± 1.44
**-1**	-2.68 ± 0.65	-3.14 ± 1.62	-2.00 ± 1.18	-1.80 ± 1.39	-0.38 ± 1.11	-1.42 ± 1.41	-0.80 ± 0.37	0.75 ± 1.53
**0**	0.00 ± 0.00	0.00 ± 0.00	0.00 ± 0.00	0.00 ± 0.00	0.00 ± 0.00	0.00 ± 0.00	0.00 ± 0.00	0.00 ± 0.00
**1**	-1.26 ± 0.71	-11.57 ± 2.01	-1.00 ± 1.75	-12.60 ± 2.56	0.53 ± 0.92	-4.42 ± 1.59	3.20 ± 2.08	-7.25 ± 1.61
**2**	-1.84 ± 0.97	-7.14 ± 1.93	1.37 ± 1.46	-11.50 ± 2.92	0.38 ± 0.76	-0.28 ± 1.71	-0.40 ± 1.44	-3.33 ± 1.43
**3**	0.73 ± 0.96	-1.14 ± 1.64	1.87 ± 2.07	-8.60 ± 2.27	1.69 ± 0.63	0.71 ± 1.81	-0.20 ± 2.65	-3.08 ± 1.97
**4**	3.52 ± 0.12	-0.57 ± 1.17	3.87 ± 1.73	-7.10 ± 2.35	-0.30 ± 1.23	1.00 ± 1.13	1.20 ± 1.85	-3.50 ± 2.06
**5**	4.00 ± 0.90	0.85 ± 0.96	6.62 ± 1.40	-5.50 ± 2.32	0.61 ± 0.98	3.42 ± 0.92	1.80 ± 1.83	-5.00 ± 3.36
**6**	6.00 ± 0.81	5.57 ± 1.02	5.12 ± 3.18	-5.80 ± 2.38	2.38 ± 0.94	-0.28 ± 1.8	1.60 ± 1.63	-5.25 ± 3.17
**7**	6.63 ± 0.46	8.00 ± 0.95	7.25 ± 1.99	-5.60 ± 3.17	1.76 ± 1.47	-0.57 ± 1.89	0.00 ± 1.48	-2.33 ± 3.37
**8**	8.73 ± 0.99	10.42 ± 1.07	7.12 ± 1.87	-3.10 ± 2.45	2.00 ± 1.61	-1.00 ± 2.18	-1.00 ± 2.3	-3.25 ± 2.84
**9**	9.57 ± 1.03	11.42 ± 1.27	8.62 ± 2.00	-2.30 ± 1.73	1.30 ± 0.86	-0.42 ± 3.22	3.00 ± 0.84	-2.08 ± 3.37
**10**	11.42 ± 1.03	13.42 ± 1.70	9.12 ± 1.99	-2.20 ± 2.17	1.53 ± 1.62	0.57 ± 2.96	4,20 ± 1.69	-2.08 ± 3.40
**11**	12.47 ± 1.20	18.14 ± 1.42	13.00 ± 2.31	-0.60 ± 2.20	2.46 ± 1.41	3.85 ± 2.96	1.20 ± 2.63	-2.45 ± 4.35
**12**	15.21 ± 1.16	19.14 ± 1.06	15.87 ± 1.93	-0.90 ± 2.51	2.92 ± 1.65	3.14 ± 2.68	4.00 ± 2.65	0.90 ± 3.67
**13**	17.00 ± 0.90	22.28 ± 0.42	16.50 ± 2.08	-0.10 ± 1.22	1.46 ± 1.77	1.00 ± 3.34	2.80 ± 2.92	-2.27 ± 3.86

**14**	19.63 ± 1.29	21.42 ± 1.21	17.75 ± 2.56	3.80 ± 2.81	1.07 ± 2.00	5.00 ± 3.46	6.60 ± 1.12	1,30 ± 3.94

Supplementary Table S8. Changes in core temperature (°C) before and after a single intracerebroventricular injection of urocortin 2

Time (min)	3-month control	3-month Ucn2	6-month control	6-month Ucn2	12-month control	12-month Ucn2	18-month control	18-month Ucn2
**-30**	-0.10 ± 0.09	-0.25 ± 0.10	-0.00 ± 0.03	-0.02 ± 0.05	0.04 ± 0.03	0.01 ± 0.04	0.05 ± 0.05	0.02 ± 0.01
**-20**	-0.10 ± 0.05	-0.14 ± 0.07	-0.02 ± 0.02	-0.04 ± 0.03	0.00 ± 0.02	0.00 ± 0.03	-0.03 ± 0.04	0.03 ± 0.04
**-10**	-0.01 ± 0.05	-0.07 ± 0.05	-0.00 ± 0.03	0.00 ± 0.01	0.01 ± 0.02	-0.01 ± 0.02	0.00 ± 0.05	0.00 ± 0.02
**0**	0.00 ± 0.00	0.00 ± 0.00	0.00 ± 0.00	0.00 ± 0.00	0.00 ± 0.00	0.00 ± 0.00	0.00 ± 0.00	0.00 ± 0.00
**10**	0.03 ± 0.05	0.10 ± 0.03	0.04 ± 0.03	0.07 ± 0.03	-0.04 ± 0.02	0.10 ± 0.04	-0.06 ± 0.05	0.13 ± 0.08
**20**	0.05 ± 0.05	0.18 ± 0.06	0.04 ± 0.04	0.14 ± 0.04	-0.02 ± 0.04	0.21 ± 0.05	-0.03 ±0.07	0.15 ± 0.10
**30**	0.01 ± 0.04	0.25 ± 0.08	0.00 ± 0.05	0.20 ± 0.05	-0.00 ± 0.02	0.26 ± 0.07	0.03 ± 0.06	0.18 ± 0.05
**40**	0.06 ± 0.04	0.32 ± 0.09	0.01 ±0.05	0.26 ± 0.06	-0.02 ± 0.03	0.31 ±0.08	-0.01 ± 0.07	0.22 ± 0.08
**50**	0.03 ± 0.05	0.43 ± 0.08	-0.04 ± 0.06	0.31 ± 0.07	-0.01 ± 0.03	0.39 ±0.09	-0.01 ± 0.08	0.28 ± 0.09
**60**	-0.01 ± 0.03	0.56 ± 0.06	-0.03 ± 0.06	0.41 ± 0.06	0.01 ± 0.04	0.46 ± 0.10	-0.13 ± 0.04	0.35 ± 0.10
**70**	0.00 ± 0.06	0.64 ± 0.06	-0.04 ± 0.05	0.48 ± 0.06	-0.01 ± 0.05	0.51 ±0.11	-0.11 ±0.04	0.43 ± 0.11
**80**	0.03 ±0.04	0.65 ± 0.06	-0.08 ± 0.05	0.56 ± 0.07	-0.06 ± 0.05	0.55 ± 0.11	-0.01 ± 0.07	0.52 ±0.11
**90**	-0.01 ± 0.07	0.64 ± 0.06	-0.06 ±0.05	0.63 ± 0.07	-0.05 ± 0.06	0.62 ±0.12	0.00 ± 0.08	0.60 ± 0.10
**100**	0.10 ± 0.06	0.67 ± 0.07	-0.10 ± 0.06	0.67 ± 0.06	-0.08 ±0.06	0.68 ± 0.12	-0.03 ± 0.06	0.75 ± 0.11
**110**	0.05 ± 0.08	0.67 ± 0.09	-0.07 ± 0.06	0.73 ± 0.07	-0.01 ± 0.06	0.78 ±0.12	-0.03 ± 0.06	0.83 ± 0.12
**120**	0.08 ± 0.08	0.7 ± 0.10	-0.07 ± 0.06	0.79 ± 0.08	-0.01 ±0.06	0.80 ± 0.13	-0.10 ± 0.06	0.88 ± 0.14
**130**	0.05 ± 0.08	0.71 ± 0.11	-0.10 ± 0.07	0.80 ± 0.08	0.00 ± 0.07	0.86 ± 0.13	-0.13 ± 0.08	0.98 ± 0.15
**140**	0.06 ± 0.11	0.70 ± 0.12	-0.12 ± 0.07	0.81 ± 0.09	-0.01 ±0.08	0.89 ± 0.14	-0.06 ± 0.11	1.07 ± 0.17
**150**	0.13 ± 0.10	0.70 ± 0.12	-0.11 ± 0.07	0.83 ± 0.09	-0.04 ± 0.07	0.94 ± 0.13	-0.03 ± 0.12	1.18 ± 0.17
**160**	0.20 ± 0.10	0.68 ± 0.14	-0.04 ±0.09	0.83 ± 0.09	-0.03 ± 0.07	0.96 ± 0.12	0.03 ± 0.13	1.22 ± 0.18
**170**	0.35 ± 0.10	0.70 ± 0.16	-0.01 ± 0.09	0.82 ± 0.08	-0.01 ±0.06	0.98 ± 0.13	0.01 ± 0.13	1.28 ± 0.17
**180**	0.38 ± 0.13	0.68 ± 0.17	0.04 ± 0.10	0.83 ± 0.09	-0.01 ±0.07	1.00 ± 0.13	0.06 ± 0.14	1.30 ± 0.07

Supplementary Table S9. Changes in oxygen consumption (ml/kg/min) before and after a single intracerebroventricular injection of urocortin 2

Time (min)	3-month control	3-month Ucn2	6-month control	6-month Ucn2	12-month control	12-month Ucn2	18-month control	18-month Ucn2
**-30**	0.21 ± 0.33	-0.12 ± 0.61	0.68 ± 0.25	0.58 ± 0.40	0.06 ± 0.43	-1.79 ± 1.21	0.39 ± 0.56	0.36 ± 0.58
**-20**	0.35 ± 0.03	-0.03 ± 0.36	0.12 ± 0.30	0.18 ± 0.43	0.39 ± 0.40	-1.01 ± 0.49	0.24 ± 0.75	0.41 ± 0.36
**-10**	0.62 ± 0.25	0.49 ± 0.34	0.24 ± 0.22	-0.00 ± 0.18	0.21 ± 0.28	-0.25 ± 0.40	-0.06 ± 0.45	0.30 ± 0.40
**0**	0.00 ± 0.00	0.00 ± 0.00	0.00 ± 0.00	0.00 ± 0.00	0.00 ± 0.00	0.00 ± 0.00	0.00 ± 0.00	0.00 ± 0.00
**10**	-0.05 ± 0.24	0.62 ± 0.40	0.08 ± 0.59	0.36 ± 0.30	0.29 ± 0.23	0.55 ± 0.20	-0.56 ± 0.27	0.86 ± 0.67
**20**	-0.91 ± 0.20	1.03 ± 0.52	0.38 ± 0.86	0.84 ± 0.67	0.23 ± 0.30	0.81 ± 0.46	-0.11 ± 0.59	1.06 ± 0.63
**30**	-0.62 ± 0.27	1.47 ± 0.61	0.04 ± 0.49	0.66 ± 0.55	0.06 ± 0.32	1.03 ± 0.68	-0.39 ± 0.41	0.65 ± 0.49
**40**	-0.51 ± 0.55	1.07 ± 0.70	0.18 ± 0.40	1.12 ± 0.59	0.38 ± 0.39	1.20 ± 0.65	-0.86 ± 0.56	0.93 ± 0.46
**50**	-0.36 ± 0.56	0.68 ± 0.59	0.00 ± 0.19	1.11 ± 0.60	-0.06 ± 0.45	1.66 ± 0.56	-0.52 ± 0.47	0.93 ± 0.43
**60**	-1.32 ± 0.49	1.12 ± 0.71	-0.04 ± 0.50	1.22 ± 0.53	-0.28 ± 0.37	1.77 ± 0.66	-1.36 ± 0.49	1.84 ± 0.35
**70**	-1.69 ± 0.43	0.83 ± 0.77	-0.06 ± 0.44	1.68 ± 0.51	-0.07 ± 0.45	1.64 ± 0.55	-1.38 ± 0.42	1.73 ± 0.62
**80**	-1.93 ± 0.44	0.12 ± 1.00	-0.20 ± 0.52	1.75 ± 0.32	-0.03 ± 0.52	2.24 ± 0.81	-1.80 ± 0.75	1.62 ± 0.59
**90**	-2.05 ± 0.46	-0.69 ± 0.84	-0.22 ± 0.25	1.91 ± 0.50	-0.38 ± 0.53	2.42 ± 0.87	-0.82 ± 0.66	1.90 ± 0.60
**100**	-2.26 ± 0.45	-0.97 ± 0.97	-0.24 ± 0.40	1.83 ± 0.56	-0.77 ± 0.52	1.96 ± 0.71	-0.58 ± 0.35	2.62 ± 0.53
**110**	-2.08 ± 0.55	-1.39 ± 0.72	-0.28 ± 0.46	1.81 ± 0.65	-0.51 ± 0.54	1.98 ± 0.67	-0.26 ± 0.58	2.49 ± 0.68
**120**	-1.89 ± 0.78	-1.37 ± 0.78	-0.88 ± 0.39	1.98 ± 0.72	-0.60 ± 0.42	2.35 ± 0.71	-0.50 ± 0.79	2.44 ± 0.53
**130**	-2.30 ± 0.64	-1.32 ± 0.80	-0.70 ± 0.40	1.92 ± 0.69	-0.54 ± 0.57	2.05 ± 0.70	-0.69 ± 0.61	2.31 ± 0.47
**140**	-2.55 ± 0.60	-1.17 ± 0.90	-0.56 ± 0.40	1.97 ± 0.58	-0.44 ± 0.61	1.69 ± 0.53	-0.53 ± 0.45	2.57 ± 0.34
**150**	-1.97 ± 0.76	-1.97 ± 0.84	-0.84 ± 0.48	1.96 ± 0.68	-0.25 ± 0.69	2.01 ± 0.73	-0.76 ± 0.49	2.90 ± 0.27
**160**	-1.82 ± 0.61	-2.12 ± 0.77	-0.32 ± 0.53	1.66 ± 0.61	-0.55 ± 0.69	1.59 ± 0.70	-0.39 ± 0.36	3.18 ± 0.45
**170**	-2.21 ± 0.39	-2.61 ± 0.72	-0.42 ± 0.72	1.18 ± 0.69	-0.44 ± 0.55	1.78 ± 0.71	0.06 ± 0.56	2.86 ± 0.32
**180**	-1.64 ± 0.22	-2.76 ± 0.74	-0.12 ± 0.48	1.24 ± 0.63	-0.55 ± 0.51	1.93 ± 0.75	0.26 ± 0.68	3.44 ± 0.48
